# LipidSpace: Simple Exploration, Reanalysis, and Quality Control of Large-Scale Lipidomics Studies

**DOI:** 10.1021/acs.analchem.3c02449

**Published:** 2023-10-04

**Authors:** Dominik Kopczynski, Nils Hoffmann, Nina Troppmair, Cristina Coman, Kim Ekroos, Michael R. Kreutz, Gerhard Liebisch, Dominik Schwudke, Robert Ahrends

**Affiliations:** † Institute of Analytical Chemistry, University of Vienna, Vienna 1070, Austria; ‡ Forschungszentrum Jülich GmbH, Institute for Bio- and Geosciences (IBG-5), Jülich 52428, Germany; § Lipidomics Consulting Ltd., Esbo 02230, Finland; ∥ Leibniz Group “Dendritic Organelles and Synaptic Function” University Medical Center Hamburg-Eppendorf, Center for Molecular Neurobiology, ZMNH, Hamburg 20251, Germany; ⊥ RG Neuroplasticity, Leibniz Institute for Neurobiology, Magdeburg 39118, Germany; # Institute of Clinical Chemistry and Laboratory Medicine, University of Regensburg, Regensburg 93053, Germany; ∇ German Center for Infection Research (DZIF), Site Hamburg-Lübeck-Borstel-Riems, Hamburg 22297, Germany; ○ Airway Research Center North (ARCN), German Center for Lung Research (DZL), Grosshansdorf 22927, Germany; ◆ Bioanalytical Chemistry, Research Center Borstel, Borstel 23845, Germany

## Abstract

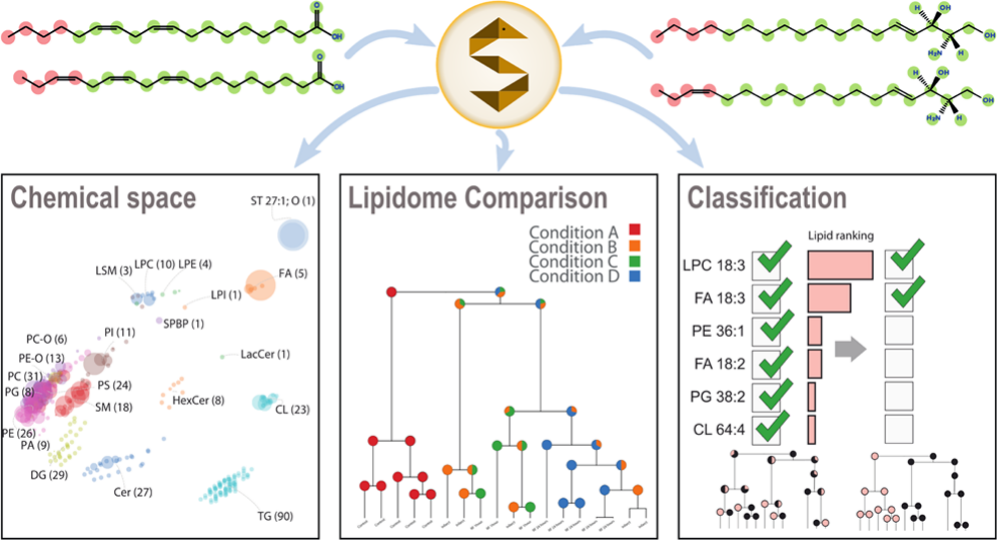

Lipid analysis gained significant importance due to the enormous range of lipid functions, e.g., energy storage, signaling, or structural components. Whole lipidomes can be quantitatively studied in-depth thanks to recent analytical advancements. However, the systematic comparison of thousands of distinct lipidomes remains challenging. We introduce LipidSpace, a standalone tool for analyzing lipidomes by assessing their structural and quantitative differences. A graph-based comparison of lipid structures is the basis for calculating structural space models and subsequently computing lipidome similarities. When adding study variables such as body weight or health condition, LipidSpace can determine lipid subsets across all lipidomes that describe these study variables well by utilizing machine-learning approaches. The user-friendly GUI offers four built-in tutorials and interactive visual interfaces with pdf export. Many supported data formats allow an efficient (re)analysis of data sets from different sources. An integrated interactive workflow guides the user through the quality control steps. We used this suite to reanalyze and combine already published data sets (e.g., one with about 2500 samples and 576 lipids in one run) and made additional discoveries to the published conclusions with the potential to fill gaps in the current lipid biology understanding. LipidSpace is available for Windows or Linux (https://lifs-tools.org).

## Introduction

Lipids contribute approximately 15–30% of the weight in an organism and serve many different functions. Their chemical and structural diversity stipulate multiple challenges in analytical chemistry and computational handling. The relatively young research field of lipidomics faces these challenges primarily for the large-scale and high-throughput characterization of lipids using mass spectrometry (MS). MS enables the analysis of hundreds of distinct lipids identified in one sample (which we refer to in the following as a lipidome) within minutes, spanning several orders of magnitude in concentration. Technological innovations are increasing throughput, stifling laboratories with more measurements requiring constant evaluation and processing. Here, we identified four main interconnected lipidomics bottlenecks, namely, the absent approach to combine quantitative results with structural information, the simple analysis of multiple lipidomes (i.e., different sets of lipids originating from other samples) with additional meta-information, the reanalysis and integration of publicly available lipidomics data, and the application of quality control methods.

Lipids can be structurally diverse, making lipidomes hard to compare when having multiple heterogeneous lipidomes or using only exact matches of the lipid species’ names/identifiers. For example, a comparison between a lipidome containing “LPC 18:0” and lacking “LPC 18:1” and a second lipidome lacking “LPC 18:0” but containing “LPC 18:1” would be counted as two distinct differences. However, both lipid species are chemically very similar. Comparisons based on structural similarity allow for a novel view and interpretation of lipidomes, even if particular lipid species are only present in some samples. So far, quantitative information is not added to these structural comparison models resulting in being heavily influenced by low abundant lipid species that do or do not appear across the sampled lipidomes. However, the quantitative lipidome data analysis remains challenging with hundreds of lipidomes and additional features or study variables (such as age, weight, or condition) associated. Many methods for lipid analysis have been published^1−3^ but do not consider these study variables, which are getting increasingly demanded. The lipidomics community is in the process of standardization in terms of controlled vocabulary, standardized nomenclature,^4,5^ data formats,^6^ reporting,^7^ or the demand of submitting both raw and result files on public repositories to meet the FAIR guidelines.^8^ The standardization will allow the community to add and reanalyze public data concerning other biological questions and enrich their data. However, the additional data and the separate handling deters researchers from adding them to their data analysis. Quality control is a multi-level process that can be embedded along the complete workflow of a lipidomics experiment. It can be conducted at the very beginning or during the data acquisition phase and consecutively during data analysis. However, without access to tools and methods that provide functions and visualizations for simple quality control (QC), QC remains laborious and time-consuming.

**Figure 1:**
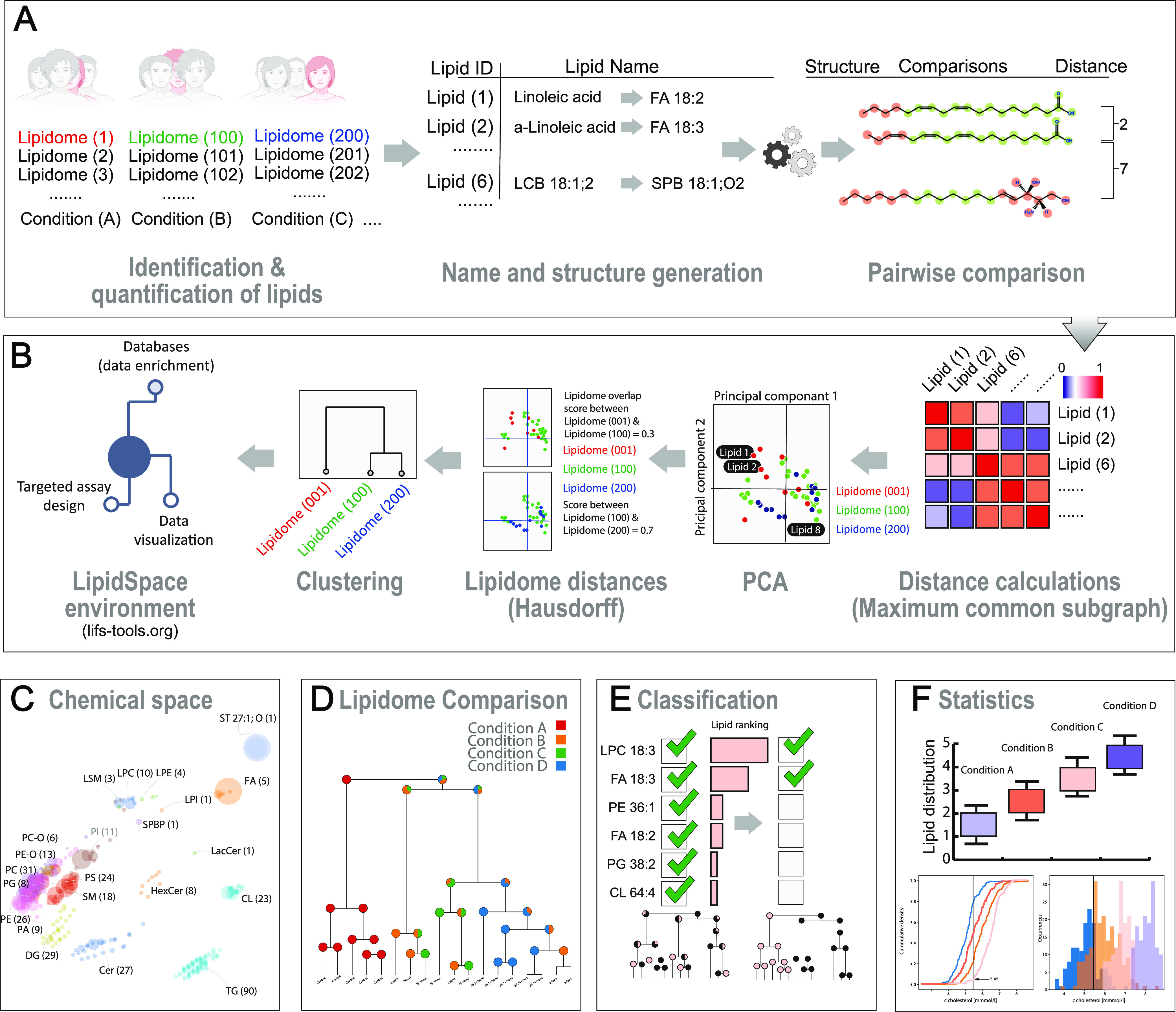
Comparative lipidome analysis and reanalysis with LipidSpace. (A) Qualitative or quantitative lipidomics data are parsed and translated to the standard shorthand nomenclature; each lipid is transformed into a chemical graph structure. (B) By applying maximum common subgraph calculations, distances are calculated between lipids. The lipidomes are visualized using principal component analysis. Determining Hausdorff distances allows a global clustering and the visualization of lipidome similarity. A rapid examination is applicable by excluding lipids, lipid classes, samples, or filtering study variables. (C) Visualizing all lipidomes within a study offers a quick grasp of the data. (D) Lipidome clustering dendrogram, including information about the study variables. (E) Classification of lipids responsible for separating lipidomes with respect to a given study variable. (F) Statistical evaluation of quantitative lipid differences across lipidomes.

Here, we introduce LipidSpace (Figure 1) for the rapid analysis of lipidomes, which addresses the above-mentioned bottlenecks by introducing the structural space and distance models. Its core feature is the structural comparison of a multitude of lipidomes. This feature compensates for the issue of missing lipids over different samples by searching for the most similar lipid counterparts for a pairwise lipidome comparison. An interactive, comprehensive view of the structural similarities between each pair of lipidomes is provided. It copes with lipidome data sets containing thousands of lipids in large-scale experiments and hundreds of samples (Figure 1A). To create such an engine, we applied the maximum common subgraph (MCS) approach in combination with the Jaccard index to determine a pairwise similarity of lipid species based on the molecular structure (see Figure 1A). Our consistent definition of a structural space that embeds lipids based on their structural features allows LipidSpace to connect all samples hierarchically (Figure 1B–D). The graphical user interface allows one to quickly adjust the computation of the structural space models by browsing through their visualizations (Supporting Figure S1). Sample-associated study variables (such as body mass index, tissue, and condition) can be added to an analysis for combined exploration. The built-in feature analysis module assists in determining which lipids exert the highest effect in distinguishing between individual study variables or combinations thereof (Figure 1E). The statistics module produces ready-to-use result figures (Figure 1F). We evaluated LipidSpace on several aspects to ensure its performance, correctness, and robustness (“the Methods section”). For all our real-size experiments (up to 1000 lipidomes), the computation time remained below 5 s (Table 1). This performance allows the application of LipidSpace in automated pipelines for real-time computation.

**Table 1:** Time Benchmark on the Lipidome Distance Model Computation in LipidSpace^a^

work	organism/tissue	#of lipidomes	#of lipids	comp. time (s)
Ejsing et al.^26^	yeast	8	248	0.082
Ishikawa et al.^27^	human plasma	60	230	0.099
Sales et al.^28^	human plasma	71	274	0.124
Carvalho et al.^29^	fruit fly/mult. tissues	12	350	0.144
Fitzner et al.^30^	mouse brain	31	616	0.184
Eggers et al.^31^	human lung	30	557	0.213
Peng et al.^32^	human/mouse platelet	60	596	0.318
Saw et al.^33^	human plasma	359	273	1.052
Wolrab et al./RC-HR	human plasma	550	366	2.065
Wolrab et al.^34^/SFC-HR	human plasma	854	200	2.068
Wolrab et al.^34^/4 comb. studies	human plasma	2499	577	48.665

a The two major measures responsible for the computation are the number of lipidomes and the number of lipids within the global lipidome (union of all lipidomes within an analysis). For all conducted experiments on real-size data from the literature, the computation time remained below 5 s, making the utilization of LipidSpace feasible for real-time applications and automated pipelines. The benchmark comprises all steps from data import up to the computation of the global lipidome distance model. The data import and rendering time of the GUI tiles and canvases needs to be taken into account here.

## Methods

LipidSpace is designed as a workstation application suitable for running on a regular office computer without access to a dedicated computing infrastructure. It is written in C++ using the Qt (https://qt.io) library for the graphical user interface (GUI). We compiled LipidSpace for the operating systems Microsoft Windows and Linux. All binaries and the source code are available via https://lifs-tools.org and from the GitHub repository at https://github.com/lifs-tools/lipidspace. The source code is published under the liberal MIT open-source license.

### Data Import

LipidSpace fully supports the current lipid shorthand nomenclature.^9^ It utilizes the C++ library of Goslin^4,5^ to parse and standardize lipid names. Lipids with lipid names of no known lipid name dialect cannot be imported. Several table formats are supported for the import, such as flat or pivot tables where lipid content is stored row-wise and the sample content column-wise or vice versa. Additionally, files in mzTab-M format^6^ containing lipidomics data can be imported, too. In general, these tables need to provide at least information on which lipid species were measured with which intensity in which sample. Here, intensity may be arbitrary units, relative concentrations (e.g., mol %), or physical units such as concentration in nmol/mL. All current lipid search engines (such as LipidXplorer,^10^ LDA,^11^ or MS-DIAL^12^) provide identification and quantification of data tables in either of the mentioned formats. Their output can be directly imported into LipidSpace.

### Determining the Similarity between Lipid Molecules

A core processing step within LipidSpace is comparing two different lipid classes resulting in a distance value. Recent research proposes feature-based comparisons for arbitrary molecules^13^ or more lipid-specific SMILES-based sequence matching for lipids, as introduced by Marella et al.^3^ However, we want to ensure that comparing two molecules always obtains the same result, even when providing them in different, non-canonical representations. To overcome this challenge, we decided to utilize the maximum common subgraph (MCS) approach^14^ in combination with the Jaccard index^15^ because molecules are best modeled as three-dimensional graph structures. The MCS approach satisfies the symmetry property of equivalence relations. That is, the distance between two lipid structures being compared with each other remains the same no matter in which order both lipids are compared: dist(A, B) = dist(B, A). Although the general problem of finding a maximum common induced subgraph is a time-consuming task, and the computation time grows exponentially with the number of considered molecules,^14^ we exploited several properties of lipids to make this approach feasible again. For instance, lipid classes are primarily structured into a fixed sub-structure (that is, the backbone and headgroup) and variable structure, for instance, the fatty acyl (FA) chains or long-chain bases (LCB) with, e.g., changing chain length and double bond numbers and positions. LipidSpace supports at the moment 131 different lipid classes, and a list of all supported lipid classes is provided in the Supporting Section S.1. Here, we claim that two different lipid species are aligned concerning their headgroup and backbone where applicable. Therefore, we can precompute the MCS for the head groups of each lipid class pair utilizing the largest weight common subtree embeddings (LaWeCSE) algorithm introduced by Droschinsky et al.^16^ This algorithm ensures the required symmetry of an MCS approach. Depending on the user-defined mode, the FAs and LCBs of two different lipids are pairwisely compared in real time, either considering their stereo-specific numbering (sn) order or selecting the best match among all permutations. When no specific distinction between fatty acyl chains and long-chain bases is necessary, we refer to them as carbon chains. One challenge is to align two carbon chains, especially when their double bond positions are not defined, for instance, FA 16:1 vs FA 18:2. Unless both carbon chains contain the exact double bond positions, our strategy is to treat these double bonds as mismatches (consider Section’ Correctness of Double Bond Comparisons). When lipid information is only available on the species level (e.g., PC 34:2), thus lacking individual carbon chain lengths, we treat these lipids as if they contain only one carbon chain. For any two lipids on different lipid information levels (e.g., one on a structurally defined level, one on a species level), we align both information levels by decreasing the higher to the lower. We also consider additional functional groups attached to the carbon chains (e.g., COOH, OH, oxo, etc.). Once the number of shared atoms and atom bonds is determined between two lipids, we compute the distance between lipid species as 1—similarity, where the similarity, the Jaccard index, is defined as the ratio between shared elements (intersection) and all elements (union, Supporting Figure S2). This ensures that the method forms an equivalence relation, which is mandatory for the robustness and correctness of the consecutive methods. We manually picked random lipids and counted their maximum common subgraphs and distance values to validate that the algorithm produced correct results.

### Creating the LipidSpace Model for One Lipidome

Having set up a robust method to compute a scalar distance between two lipid species, the second step is to form a model representing the relation of all lipids within a sample/lipidome to each other. Therefore, we first prepare a list of all distinct lipid species among all lipidomes considered for analysis. Next, we compute all pairwise distances between these lipid species, resulting in an *n* × *n* matrix where *n* denotes the number of lipid species. Afterward, we perform a principal component analysis (PCA) on the complete matrix. We decided to use an implementation of the Lanczos method^17^ [https://github.com/mrcdr/lambda-lanczos] to compute only the first *n*’ *< n* principal components to reduce overall computation time. We are utilizing the openBLAS [https://www.openblas.net/] library for fast matrix multiplication, providing fast basic linear algebra operations. By default, the lipid Space model contains seven principal components (best trade-off between accuracy and performance). However, this property can be set by the user manually. Since the result includes a union set of lipids from all lipidomes within the analysis, we denote it as the global lipid space model. The primary visualization of this model uses, by default, the first and second principal components as *x* and *y* Cartesian coordinates. Further, we visualize each lipidome with its respective subset of lipids and their abundances (Figure 1C; or Supporting Figure S1, top right). In LipidSpace, the user can alternate the visualization by changing the mapping of the principal components onto the *x* and *y* dimensions. The visualizations (Supporting Figure S3) themselves provide browsing functions for simultaneous moving and zooming, affecting each PCA plot simultaneously with regular mouse controls.

### Setting All Lipid Space Models in Relation to Each Other

Once all lipid space models are derived from the global model, the user can continue the analysis. Either proceed with the qualitative data or add the lipid quantities to the analysis. By default, quantities are included and may be switched off at any time. The objective is to put all lipid spaces into relation with each other. Two lipidomes may be very heterogeneous and contain two sets of lipid species with little overlap. However, to avoid basing the distance of two lipidomes purely on the common lipid species, we applied the Hausdorff distance^18^ (using a fast implementation^19^), which measures the distance of two subsets of elements of a metric space. When considering quantities, the abundances for a lipidome are normalized concerning the variance of the first PC of its model and added to the model. Applying pairwise distance computations on *m* lipidomes results in an *m* × *m* distance matrix. In the last step, we calculate an agglomerative hierarchical clustering, either using single linkage, unweighted average linkage, or complete linkage clustering. By default, unweighted average linkage clustering is selected. The resulting hierarchical clustering, which we refer to as the global lipidome distance model, is visualized by an interactive dendrogram plot (Supporting Figure S4). The interactive visualization contains functions for a rapid and convenient examination of the lipidomes. For instance, if only a sub-branch of the complete set of lipidomes should be examined, the user can select the corresponding sub-branch and start a new analysis on all lipidomes contained within it. All visualizations can be exported in PDF format.

### Feature Analysis and Selection

LipidSpace can operate with sample-related study variables, lipids, and their abundances. Here, we distinguish between categorical study variables, such as a smoker with values yes or no, or condition, with values control or perturbed, and numerical study variables, such as age, weight, body mass index, cholesterol concentration, etc. If such information is available, the lipidome distance dendrogram adds it to every branch. We apply the Kolmogorov–Smirnov (KS) statistic to find the best value. When drawing the cumulative density functions (CDF) for both sets, we search for the position of the largest difference between both CDFs (Supporting Figure S5). We assign this value as the best separation value. Each inner node has a left and right branch in the dendrogram. For both branches, a pie chart shows the relative distribution of values within the sub-branch according to the best KS separation value (Supporting Figure S5).

In the following, we will denote lipids as features to find the most important lipids acting on a specific study variable. Having a set of lipids, an exhaustive search for the optimal subset is not applicable since a set’s number of possible subsets grows exponentially with the number of elements contained in it. Nowadays, most modern studies cover 100s of lipids. We, therefore, applied an efficient, fast heuristic sequential forward selection (SFS) of features.^20^ SFS starts with zero selected features. The first step assesses which single lipid best describes the study variable based on a classification or regression model. We used multiple linear regression with the Akaike Information Criterion (AIC)^21^ to assess model performance. The lower the AIC, the better the performance of the regression model. Let *n* be the total number of features. Therefore, in the first step, *n* models are computed. From these *n* models, the model with the lowest AIC is chosen. In the second round, all two-feature subsets with the feature from the previous round are examined. In the third round, all three-feature subsets with the two features from the previous round are examined, and so on. After *n* steps, we obtain a well-performing feature subset for each round (representing the number of features). In the last step, we pick the model performing the best overall rounds. The computation time is quadratic with respect to *n*. To reduce computational time and avoid overfitting with too many features, we perform only the first √*n* steps.

### Statistics

LipidSpace contains a statistic module comprising several aspects of the imported data. Based on the provided study variables, bar plots, histograms, and box plots visualize the distribution of lipidomes (or lipids, respectively) with respect to their lipid quantities. For all bar plots and box plots, the underlying source data can be added to the plot as a scatter plot above the boxes. The box plots on the lipid level also offer the visualization of statistical results. Box plots on the lipidome level contain either a *t*-test or ANOVA (depending on the number of distinct values within a study variable). The lipidome histogram contains an accuracy measure for the capability of the selected lipids to describe the selected study variable. When having a nominal study variable with only two values (e.g., knockout vs wild type), a receiver operating characteristic (ROC) curve figure is provided. It illustrates how well a selection of lipids among all lipidomes can separate both conditions from each other. Another quality control measure is the distribution of coefficients of variation (CV) for each lipid again based on the provided study variables. For instance, data sets from two different experiments/studies can quickly be checked if having a similar CV distribution (if expected) or not. Another figure contains a *p*-value distribution plot with an adjustable statistical test based on a chosen nominal study variable. An equally distributed *p*-value histogram might indicate either no regulation between the groups of the chosen study variable or, e.g., a misconducted experiment where a preceded perturbation did not occur. A more advanced statistical figure is the volcano plot, which only appears when a nominal study variable with only two categories is selected. All these statistics are also available in other statistics programs such as MetaboAnalyst, LipidSuite, or Lipid Mini-On.^22−24^ However, we included them into LipidSpace for user convenience to offer an all-in-one solution.

### Quality Control in LipidSpace

For QC, visualizations and statistic figures described in the previous section are implemented in LipidSpace. Further, a built-in QC function tests during data import if the loaded data conforms to Benford’s law^25^ and informs the user with additional details if not. According to the law, a big set of numbers (especially obtained from observations) over several orders of magnitude have a leading digit distribution of a reciprocal function. Lipidomics data not conforming to the law might indicate a low range of quantitative values or insufficient data imputation (if applied before). The fourth interactive built-in tutorial introduces best practice methods for QC (see the “Interactive Tutorials” section). A list of all measures and approaches that can be applied for QC in LipidSpace is available in the Supporting Section S.2.

### Evaluation of LipidSpace to Ensure Performance, Correctness, and Robustness

#### Performance

LipidSpace is implemented in C++ and utilizes highly optimized mathematics libraries for its computations. Thus, it can quickly process big data sets with over 1000 lipidomes. We tested the performance of the analysis routine, where the lipid spaces for each lipidome and the global lipidome distance model were computed, excluding rendering times of the Graphical User Interface. Our testing platform was a standard laptop (Lenovo Thinkpad X1 Carbon, Intel i7 1.8 GHz octa-core laptop, 16 GB main memory). The results are presented in Table 1. The overall computation time depends on both the number of lipidomes and the total distinct number of lipid species. For instance, the computational complexity of computing the PCA is cubic with respect to the number of provided distinct lipid species (O(*n*^2^)). On the other hand, the complexity of computing the Hausdorff distances and the resulting hierarchical clustering is quadratic in the number of lipid species (O(*n*^2^)) and cubic to the number of provided lipidomes (O(*m*^3^)). However, many expensive steps within the computational pipeline can be easily parallelized, such as computing the pairwise lipid or Hausdorff distances. By utilizing highly optimized mathematics libraries and fast implementations, we achieved analysis times between 0.1 s for 8 lipidomes and 229 lipids and up to 48 s for 2499 lipidomes with 577 lipids. Nevertheless, for all experiments with <1000 lipidomes, the computation time remained below 5 s (Table 1). This allows an application of LipidSpace in automated pipelines for real-time computation.

#### Correctness of Double Bond Comparisons

Our second experiment focuses on the situation when lipids with fatty acyl chains or long-chain bases are provided without any specific double bond (DB) position. We, therefore, extracted all lipids from databases such as LIPID MAPS and SwissLipids on the highest structural resolution level, containing explicit information about all double bond positions on their carbon chains. We extracted all carbon chain information from each database individually. Next, we calculated a pairwise comparison of all carbon chains for each database to determine the probability of two arbitrary carbon chains having *x* overlapping (at the same position) double bonds. We checked counting the DB positions from both ends, from the carbonyl carbon group (forward) and from the methyl group (ω/backward). For instance, when comparing the fatty acyl chains linoleic acid/FA 18:2(9Z,12Z) and arachidonic acid/FA 20:4(5Z,8Z,11Z,14Z) with each other, their double bond positions counted from the beginning (9, 12) and (5, 8, 11, 14) have no match, but two matches (ω-6, ω-9) and (ω-6, ω-9, ω-12, ω-15) when counting backward. Supporting Figure S6 illustrates the result of this experiment. For the LIPID MAPS database, a complete mismatch probability of at least 73% in both directions at 73%. Since the SwissLipids database only contains 85 distinct DB sets, the mismatch probability in the forward direction is about 64% but also over 71% in the backward direction. Therefore, when no DB position information is provided, we automatically count all DB matches as mismatches for each pairwise lipid comparison to find the maximum common subgraph. When DB positions are provided, the MCS will be computed by considering the DB positions in the forward direction. For example, a comparison of the lipids “FA 18:2” and “FA 18:1” provides 36 common/matching components (atoms and bonds) out of 39 united components in an MCS. In contrast, a comparison of “FA 18:2(9,12)” and “FA 18:1(9)” provides 38 intersecting components out of 39 united components.

#### Robustness of Structural Preservation in Higher Dimensions

The following experiment is designed to validate the robustness of LipidSpace model generation by comparing its output with published results. We created a list of 14 diacylglycerophosphocholines PC 12:0/[12−24,26]:0 to examine the spatial organization of lipids in a structural space model (Supporting Figure S7). The structural similarity between these 14 lipids was determined in a model consisting only of these lipids and within an extensive set of lipids (here 500 lipids from different categories). They preserve the sequential positioning in the shape of an arc, although slightly distorted, giving evidence that they keep their individual distances to each other even when higher dimension calculations are required.

### Interactive Tutorials

LipidSpace is equipped with four interactive tutorials on different topics guiding the user through the actual user interface (UI) by enabling only the UI controls that are necessary for the current tutorial steps. The tutorials are designed to give an introduction to (i) data import, (ii) handling of the UI for result interpretation, (iii) feature analysis, and (iv) quality control methods.

## Results and Discussion

**Figure 2:**
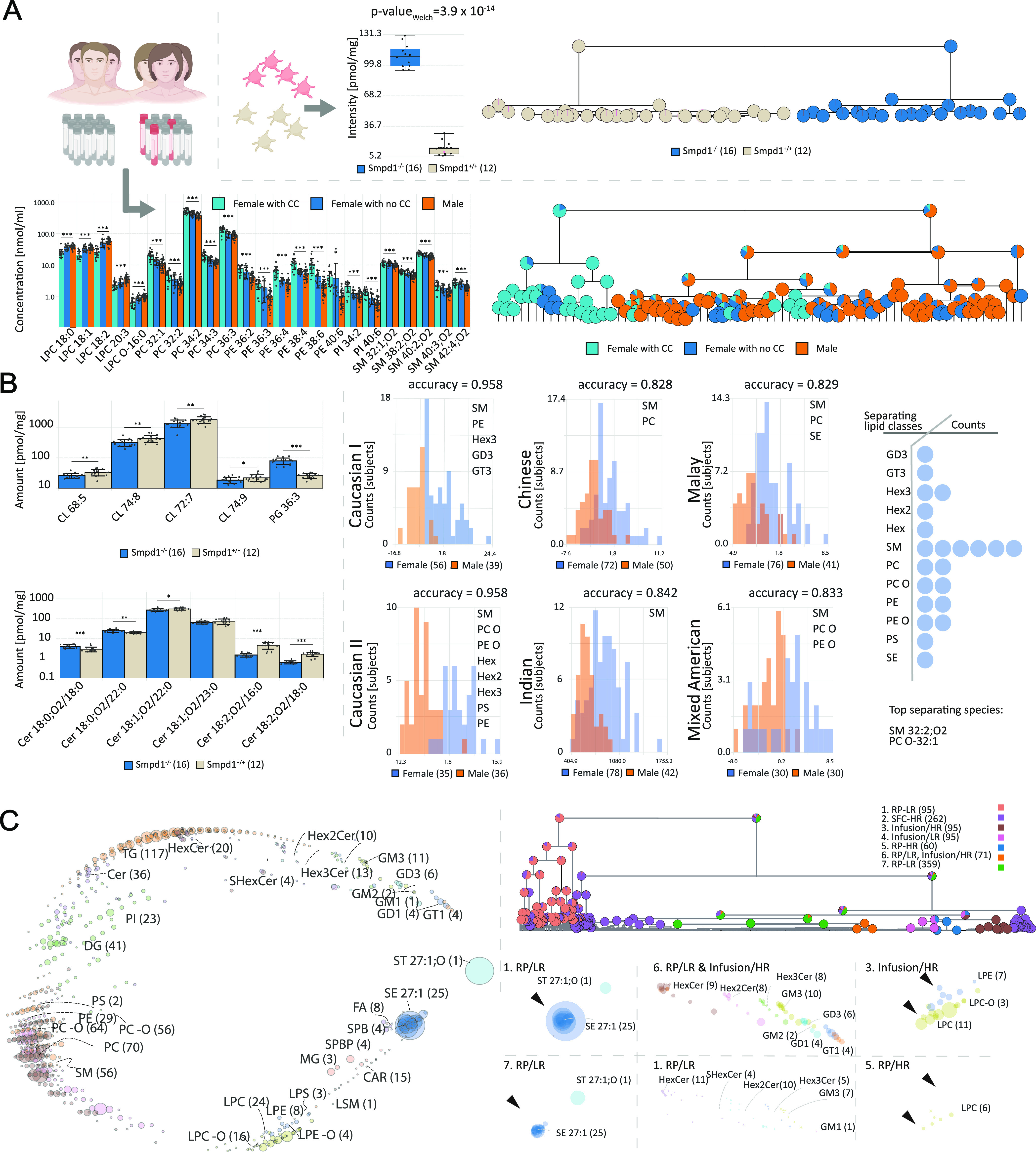
Different use cases for LipidSpace. (A) Confirmation of discoveries in published studies: (upper) LSM 18:1;O2 performs well to separate all measured samples based on their condition^32^ with an accuracy of 100%, (lower) main differences in lipid concentration of male and female (either taking contraceptives (CC) or not) plasma lipidomes^28^ caused by PC, PE, and SM lipids. 23 lipids can achieve separation of data sets with an accuracy of 93%; (B) extended analyses on published studies: (left) feature analysis on Smpd1 knockout^32^ data reveal a correlation between the increase of PG and decrease of CL and a systematic shift of double bonds for Cer 18:*x*;O2 subclass, (right) six ethnicity-dependent studies show that SM had the most lipid species involved in the gender separation models; (C) QC of seven different human plasma experiments: (left) global structural space of all 1037 samples, (top right) hierarchical clustering of all samples shows similarities across four studies (experiment 1–4 from study,^34^ ex. 5 from ref (27) 6 from ref (28), and 7 from ref (33)), (bottom right) study aggregated structural spaces reveal differences of lipid species compositions (at most on sn-position level) and concentrations among studies.

We reanalyzed open-access platelet and plasma data sets to prove LipidSpaces applicability. The reanalysis of the platelet lipidome data^32^ within acidic sphingomyelinase (Smpd1^–/–^) mouse knockout (KO) across different stimuli confirms the author’s results that LSM 18:0;OH and LSM 18:1;O2 can separate both conditions with an accuracy of 100% (Figure 2A, top). A comparison of lipid concentrations in males and females in the second study^28^ revealed significant differences mainly in the lipid classes PC, PE, and SM, confirmed by applying LipidSpace (Figure 2A, bottom), too. The third study^33^ investigated the differences in lipid compositions among three Asian ethnicities. As in the study, LipidSpace also computed that PC O-40:7 and PE O-40:7 show the best separation capabilities with a *p*-value ≪ of 0.001 (Supporting Figure S8). In the second phase, we searched for new potential mechanisms and differences in lipid concentrations within the same studies. For the first study, we revealed that the best separation result could also be achieved by PG 36:3 with 100% accuracy (Figure 2B, top left). Especially, the increase of the main PG species (Supporting Figure S9) is interesting since they are cardiolipin (CL) precursors. The dropping concentrations (Figure 2B, top left) of four major abundant CL species might indicate a reduced CL metabolism or fewer mitochondria and tentatively a mitochondrial impairment in the Smpd1-deficient platelets. Additionally, a systematic shift of double bonds for the Cer 18:*x*;O2 subclass is recognizable with Cer 18:0;O2 lipids increasing during knockout, Cer 18:1;O2 show low variation, and Cer 18:2;O2 are significantly decreasing (Figure 2B, bottom left). We can only speculate why individual Cer 18:2;O2 levels are dropping. However, with a reduced mitochondrial capacity indicated by lower CL levels, enzymes responsible for ceramide desaturation may already be reduced during proplatelet formation to lower energy consumption and stabilize signaling.^35^ Since gender differences in metabolism are still poorly defined, we reinvestigated the female lipid metabolism across ethnicities. Therefore, we compared the data from several different human plasma lipidome studies.^27,28,33,34^ We identified sphingomyelins (SM) as the main discriminators for gender-specific lipidomes (Figure 2B, right) overall studies. Further analysis of enzymatic activities of “acidic sphingomyelinase” or “sphingomyelin synthase 2” might uncover an underlying mechanism explaining the significantly higher concentrations of SM in the female plasma lipidomes. Additional results for the third study were achieved by comparing lipidomes derived from Chinese and Indian populations since their lipidomes differed the most (Supporting Figure S10). Using the feature selection function of LipidSpace, we detected that many lipids distinguishing both lipidome sets are polyunsaturated phospholipids containing ether bonds with dropping levels in the Indian subgroup. Since ether lipids are known to work as scavenger molecules of radicals, we assume that the Indian cohort experienced more oxidative stress due to the different food diets. LipidSpace supports assessing differences across several platforms regarding missing species and divergent quantities. We, therefore, evaluated in the third phase seven published human plasma lipidomics experiments within four studies^27,28,33,34^ measured with different acquisition techniques for QC. For the heterogeneity of the cohorts, we only considered samples from healthy humans within the data sets. This evaluation covers 702 different lipid species over 1037 samples (Figure 2C, left). A pure qualitative comparison (Supplementary Figure S11) displays that samples within their studies have a higher species overlap than across the studies. When adding quantitative data, the distances between samples from different studies are reduced (Figure 2C, top right), indicating that a certain consensus is achieved among all samples (because many differing lipids are of low concentration with little impact). When comparing the global lipidome with study-specific lipidomes, one can see which lipid classes are primarily present or completely absent (Figure 2C, bottom right). For instance, in some studies, sphingolipid classes are missing, while sterol esters (SE) are noticeably higher in others.

## Conclusions

To our best knowledge, we introduced LipidSpace, which is (to our best knowledge) the first tool capable of processing large-scale lipidomics experiments in a minute by examining the structural and quantitative distance of all lipidomes to each other. A fully interactive graphical user interface simplifies the lipidomes’ examination by browsing through one lipid space model, browsing several lipid space models simultaneously, or investigating the global lipid distance model. On top, the built-in feature analysis function makes it easy to search for high-impact lipids within this experiment. Selecting or deselecting lipid species, classes, categories, sample features, or even complete samples allows the user to verify the impact on these entities by quickly reanalyzing the remaining lipids. It provides multiple possibilities for quality control at several stages. The performance of LipidSpace allows it to be applied in real-time pipelines or systems such as web services. Several input file formats support makes it even easier to integrate the tool into an existing workflow. In summary, LipidSpace enables horizontal (across studies) and vertical (across species) lipidome comparisons in combination with associated study variables, as needed for analysis of clinical studies, and opens further avenues to gain insights into the lipid-specific composition and its connection to underlying cellular mechanisms.

## Data Availability

LipidSpace can be downloaded as Windows or Linux binary (no installation necessary) from our portal https://lifs-tools.org. The source code is available under the MIT license at https://github.com/lifs-tools/lipidspace.

## Supporting Information Available

List of supported lipid classes in LipidSpace; quality control measures and approaches; the graphical user interface of LipidSpace; visualization of an exemplary lipidomics space analysis (PDF)
