# Supplementary material for: LipidSpace: Simple Exploration, Reanalysis, and Quality Control of Large-Scale Lipidomics Studies
Source: Anal Chem. 2023 Oct 4;95(41):15236–44. doi: 10.1021/acs.analchem.3c02449 (PMC10585661; doi:10.1021/acs.analchem.3c02449)
Supplement: Supplementary file 1 — ac3c02449_si_001.pdf [file ac3c02449_si_001.pdf]

Supporting Information - LipidSpace: simple exploration, re-analysis, and quality control of large-scale lipidomics studies

Dominik Kopczynski[1], Nils Hoffmann[2], Nina Troppmair[1], Cristina Coman[1], Kim Ekroos[3], Michael R. Kreutz[4,5], Gerhard Liebisch[6], Dominik Schwudke[7,8,9], Robert Ahrends[1*]

[1] *Institute of Analytical Chemistry, University of Vienna, Vienna, Austria.*
[2] *Forschungszentrum Jülich GmbH, Institute for Bio- and Geosciences (IBG-5), Jülich, Germany.*
[3] *Lipidomics Consulting Ltd., Esbo, Finland.*
[4] *Leibniz Group 'Dendritic Organelles and Synaptic Function' University Medical Center Hamburg-Eppendorf, Center for Molecular Neurobiology, ZMNH, Hamburg, Germany*
[5] *RG Neuroplasticity, Leibniz Institute for Neurobiology, 39118 Magdeburg, Germany*
[6] *Institute of Clinical Chemistry and Laboratory Medicine, University of Regensburg, Regensburg, Germany.*
[7] *German Center for Infection Research (DZIF), Site Hamburg-Lübeck-Borstel-Riems, Hamburg, Germany.*
[8] *Airway Research Center North (ARCN), German Center for Lung Research (DZL), Grosshansdorf, Germany.*
[9] *Bioanalytical Chemistry, Research Center Borstel, Borstel, Germany.*
[*] *Corresponding author, email: robert.ahrends@univie.ac.at.*

Table of Contents

Supplementary Information....................2
S.1) List of supported lipid classes in LipidSpace....................2
S.2) Quality control measures and approaches....................2
Supplementary Figures....................4
References....................9

Supplementary Information

S.1) List of supported lipid classes in LipidSpace

BMP, CAR, CDPDAG, Cer, CerP, CerPE, CL, DG, DGDG, DLCL, DMPE, EPC, FA, FAHFA, FOH, Gal-Gal-Glc-Cer, Gal-GalNAc-NeuAc-Gal-Glc-Cer, Gal-GalNAc-NeuAc-NeuAc-Gal-Glc-Cer, Gal-GalNAc-NeuAc-NeuAc-NeuAc-Gal-Glc-Cer, Gal2GalNAcGlcNeuAc2Cer, Gal2GalNAcGlcNeuAc3Cer, Gal2GalNAcGlcNeuAc4Cer, Gal2GalNAcGlcNeuAc5Cer, Gal2GalNAcGlcNeuAcCer, Gal2GlcCer, GalCer, GalGalNAcGlcNeuAc2Cer, GalGalNAcGlcNeuAc3Cer, GalGalNAcGlcNeuAcCer, GalGlcNeuAc2Cer, GalGlcNeuAc3Cer, GalGlcNeuAcCer, GalNAc-NeuAc-Gal-Glc-Cer, GalNAc-NeuAc-NeuAc-Gal-Glc-Cer, GalNAc-NeuAc-NeuAc-NeuAc-Gal-Glc-Cer, GalNeuAcCer, GD1a, GD1b, GlcCer, GT1a, GT1b, GT1c, Hex2Cer, Hex3Cer, HexCer, IPC, LacCer, LCL, LHexCer, LIPC, LPA, LPA-O, LPA-P, LPC, LPC-O, LPC-P, LPE, LPE-O, LPE-P, LPG, LPG-O, LPG-P, LPI, LPI-O, LPI-P, LPIM1, LPIM2, LPIM3, LPIM4, LPIM5, LPIM6, LPS, LPS-O, LPS-P, LSM, M(IP)2C, MG, MGDG, MIPC, MMPE, NeuAc-Gal-Cer, NeuAc-Gal-GalNAc-NeuAc-NeuAc-NeuAc-Gal-Glc-Cer, NeuAc-Gal-Glc-Cer, NeuAc-NeuAc-Gal-GalNAc-NeuAc-NeuAc-NeuAc-Gal-Glc-Cer, NeuAc-NeuAc-Gal-Glc-Cer, NeuAc-NeuAc-NeuAc-Gal-Glc-Cer, PA, PA-O, PA-P, PAT16, PAT18, PC, PC-O, PC-P, PE, PE-O, PE-P, PEt, PG, PG-O, PG-P, PI, PI-O, PI-P, PIM1, PIM2, PIM3, PIM4, PIM5, PIM6, PIP, PIP2, PIP3, PS, PS-O, PS-P, SE 27:1, SHexCer, SM, SPB, SPBP, SQDG, ST 27:1;O, ST 27:2;O, ST 28:1;O, ST 28:2;O, ST 28:3;O, ST 29:1;O, ST 29:2;O, ST 30:2;O, TG

S.2) Quality control measures and approaches

Benford's law: According to number theory, the first digits of a set of numbers do not occur with equal probability when the numbers are spanning several orders of magnitude. A set of numbers violating this property (Benford's law [1]), might indicate lipid quantities of low order of magnitude or a poorly performed data imputation for missing values.

Principal component analysis for blank assessment: Blanks might indicate a good technical data acquisition when measured equally distributed throughout the complete sample measurement process. Blanks should contain a similar analyte composition and thus be quite similar. Blanks that do not cluster well together in a PCA may indicate issues in the sample acquisition process.

Coefficient of variation / Relative standard deviation: With respect to a selected nominal study variable, CV histograms of the lipids within the respective groups are plotted. CV values exceeding 25-30 % may indicate insufficient measurement of samples or individual lipids.

Comparison of structural similarity of samples on either qualitative level or quantitative level: The hierarchical dendrogram supports quality control in numerous ways. For instance, lipidomics identification results from two different laboratories can be quickly assessed for equality based on shared lipid presence. When turning off quantitative data from the dendrogram, one can see if

samples measured from different laboratories distribute randomly or if they form two separate clusters. If the latter is the case, the laboratories may have used different methods leading to identification of non-concordant lipid sets.

When working with model organisms, reference lipidome tables can be sourced from literature to compare the performance of one's own control measurements to the reference on a quantitative level. The formation of well separated clusters for both the reference samples and own control measurements (even with applied quantity normalization) may again hint at issues stemming from the pre-analytical or sample acquisition process.

Adjustable p-value distribution: When a nominal study variable is chosen with at least two categories, a p-value distribution plot is available. The type of test is adjustable (Student's t-Test, Welch's t-Test, Kolmogorov-Smirnov Test, or ANOVA for comparison of more than two categories). An equal distribution of p-values might indicate that either no regulation exists between these categories or that a preceding experiment between these categories (e.g., knockout vs. wildtype) did not succeed.

Adjustable volcano plot for nominal study variables with two categories: An enhancement of the p-value distribution is a volcano plot. Lipid quantities are being compared between both samples where their logarithmic ratio (fold change) is reported on the x-axis and their (negative logarithmic) p-value on the yaxis, often resulting in a volcano-shaped scatter plot. Again, points not exceeding any predefined limits might indicate absence of regulation between both categories or that an preceding experiment to these categories (e.g., knockout vs. wildtype) did not succeed.

Supplementary Figures

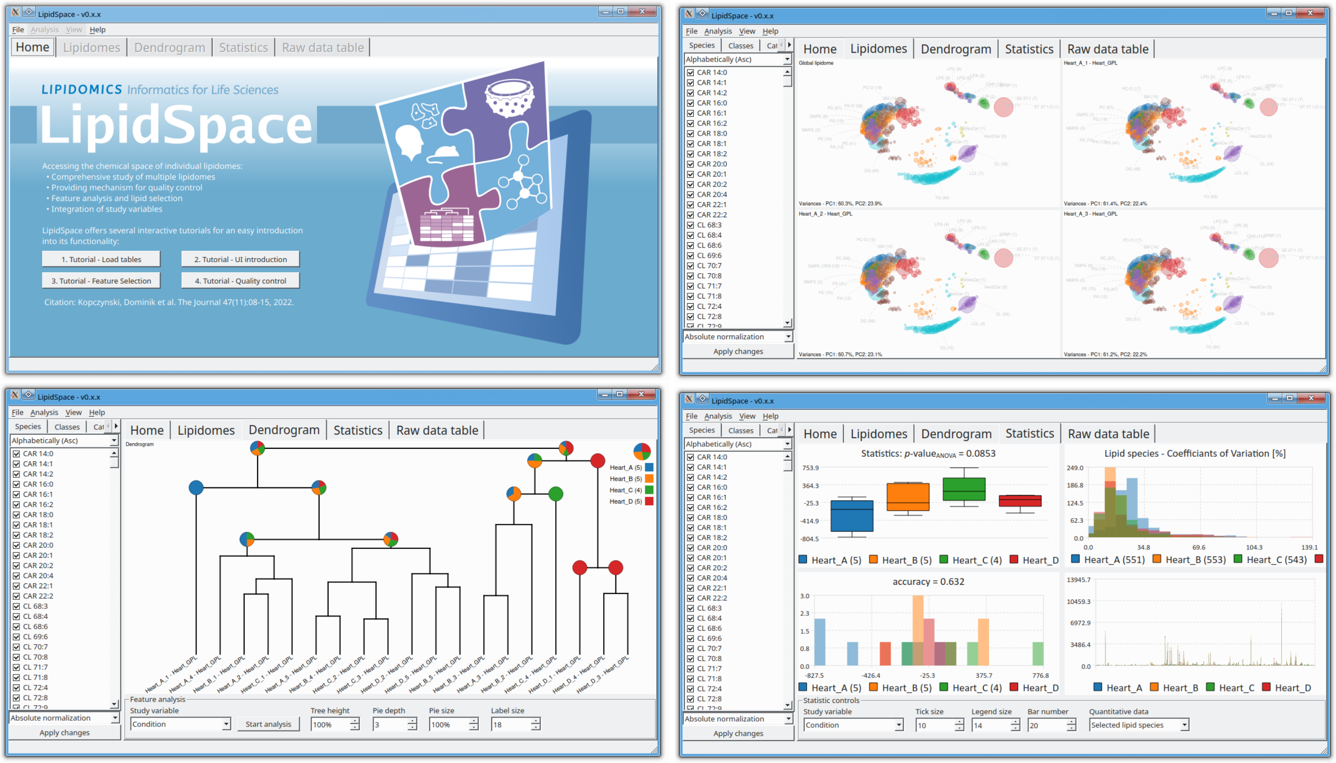

Figure S1: The graphical user interface of LipidSpace. Several visualization modules are available via the landing page with its interactive tutorials (top left), the structural lipid space models with either a global model over all lipidomes or individually for each lipidome (top right), an interactive dendrogram visualizing the hierarchical relation between all lipidomes (bottom left), and a module providing several statistics and figures for download (bottom right).

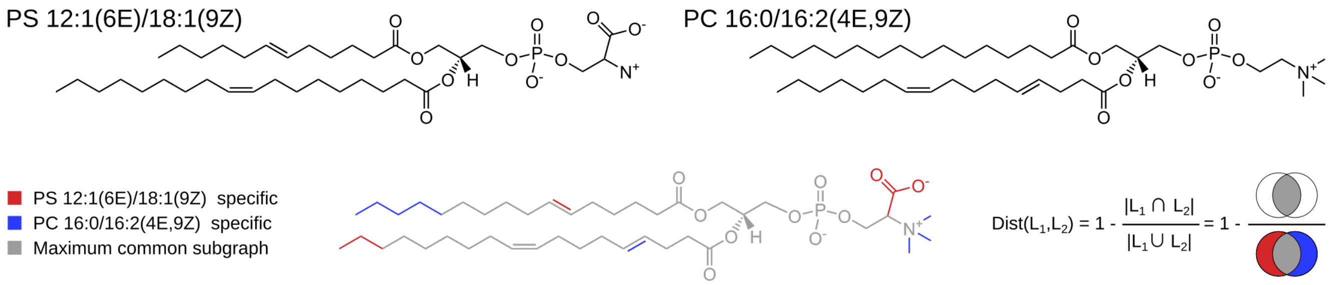

Figure S2: Maximum common subgraph between PS 12:1(6E)/18:1(9Z) and PC 16:0/16:2(4E,9Z). The MCS shares atoms and bonds (grey) and has unique structures for the first lipid (red) and the second lipid (blue). The similarity (Jaccard index) is the ratio between the shared number of elements (intersection) and all elements (union) ranging from 0 to 1. The distance between both lipids is defined as 1 – the similarity and also ranges from 0 to 1. In this example, 83 elements (atoms and bonds) are shared by 109 common elements resulting in a distance value of 0.238 without unit.

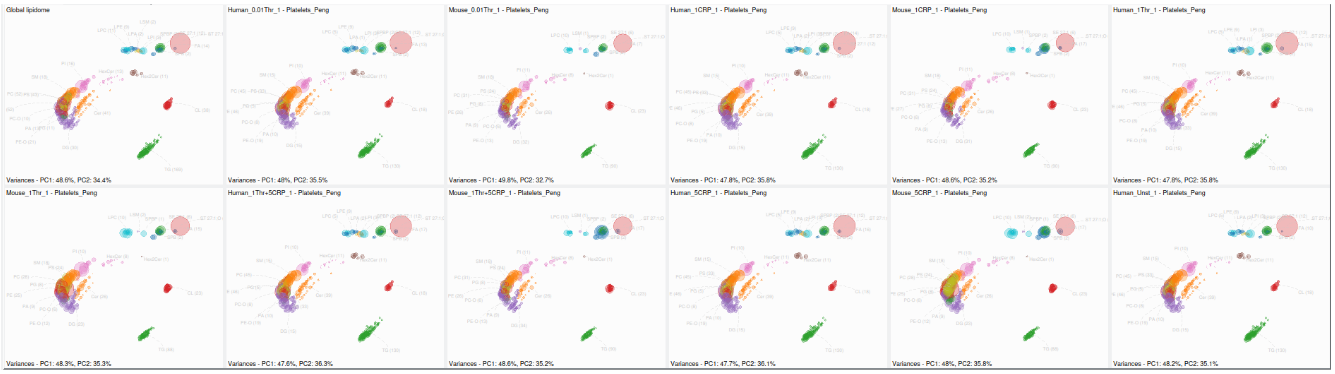

Figure S3: Visualization of an exemplary lipidomics space analysis. In total, eleven samples (six human and five mouse samples) were analyzed in this experiment resulting in eleven structural lipid space models (tiles 2 - 12) and a comprehensive global lipid space model (tile 1, top left).

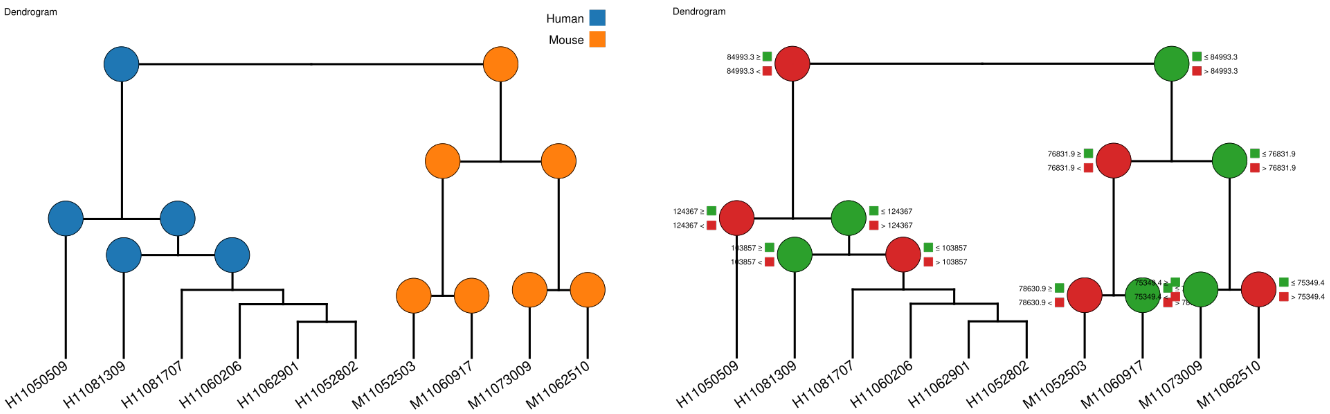

Figure S4: Visual representation of study variable values in the dendrogram. When selecting nominal study variables (left) in LipidSpace, the branches show a pie chart of lipidome distributions associated to the values in the respective subtree. Having selected a numerical study variable (right), the best separation value is computed between two connected sub-branches and the pie charts show the distribution of lipidomes having a higher or lower value. For example, here the study variable ‘cholesterol (pmol/mg protein)’ (right) is shown. The top level can be separated at the value 84993.3. The color green indicates a value less than 84993.3. For each horizontal branch, a new separation value is computed as explained in Figure S5.

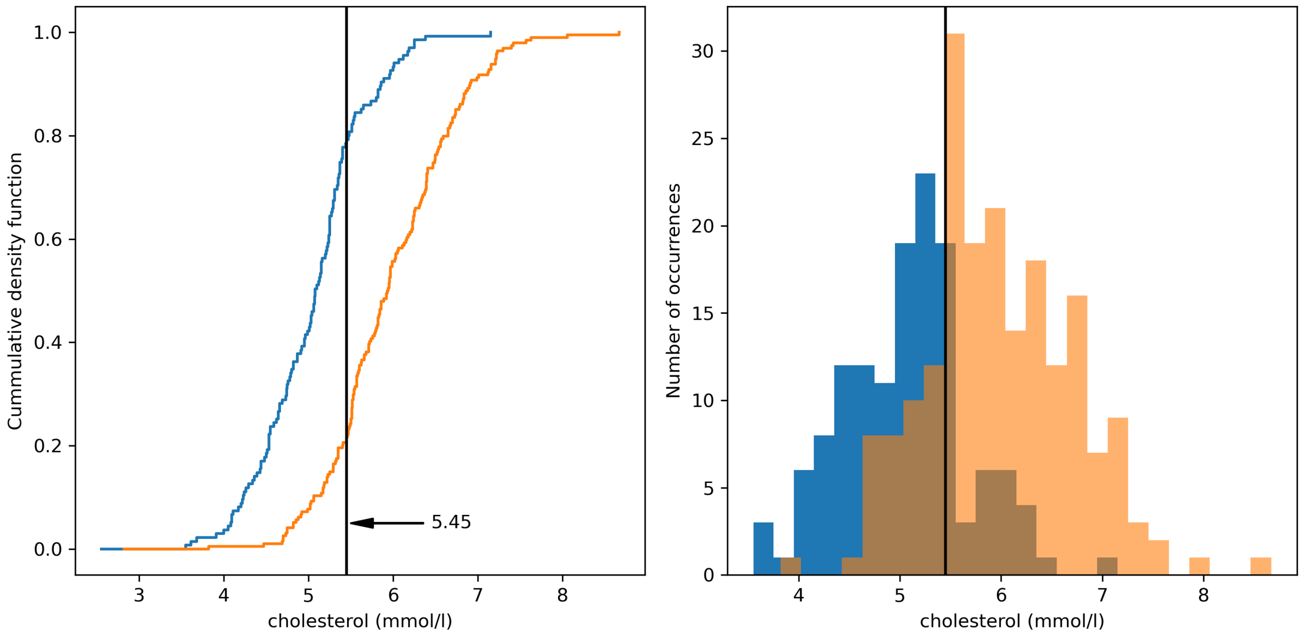

Figure S5: Determination of optimal value for separation of two numerical sets. (left) Both sets are sorted and their cumulative density function is computed, while the highest cumulative difference determines their optimal separation value. (right) A histogram illustrates the distribution of both sets. For this example, the sets were extracted from a dataset from a study on human plasma published by Saw et al. [2]. The study variable is the concentration of cholesterol (mmol/l) for 359 human samples.

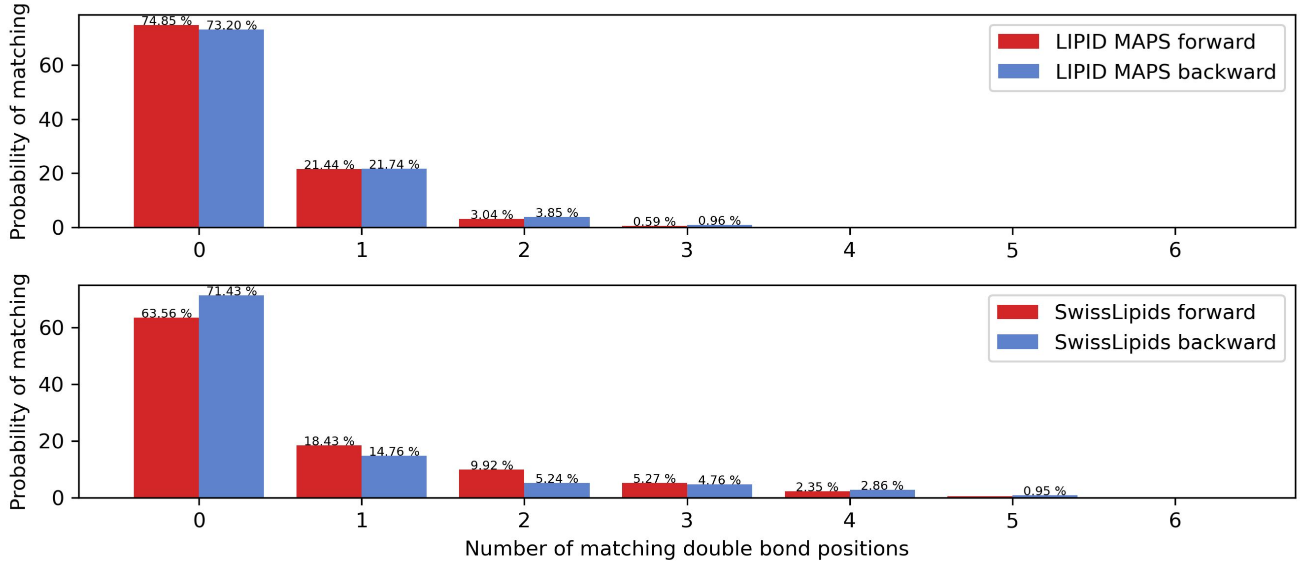

Figure S6: Probability distribution of two arbitrary carbon chains having x double bond position matches. We based our comparison on the two established lipid databases LIPID MAPS and SwissLipids. For each database, a distinct set of fatty acyl chains with at least one double bond position information was extracted. Double bond (DB) positions were compared either when starting to count from the carbonyl carbon group (forward) or from the methyl group (omega / backward). The probability that two arbitrary fatty acyl chains with a different DB composition have zero matching double bond positions is at least 73 % in both directions for the LIPID MAPS entries and between 64 % and 71 % for the SwissLipids entries.

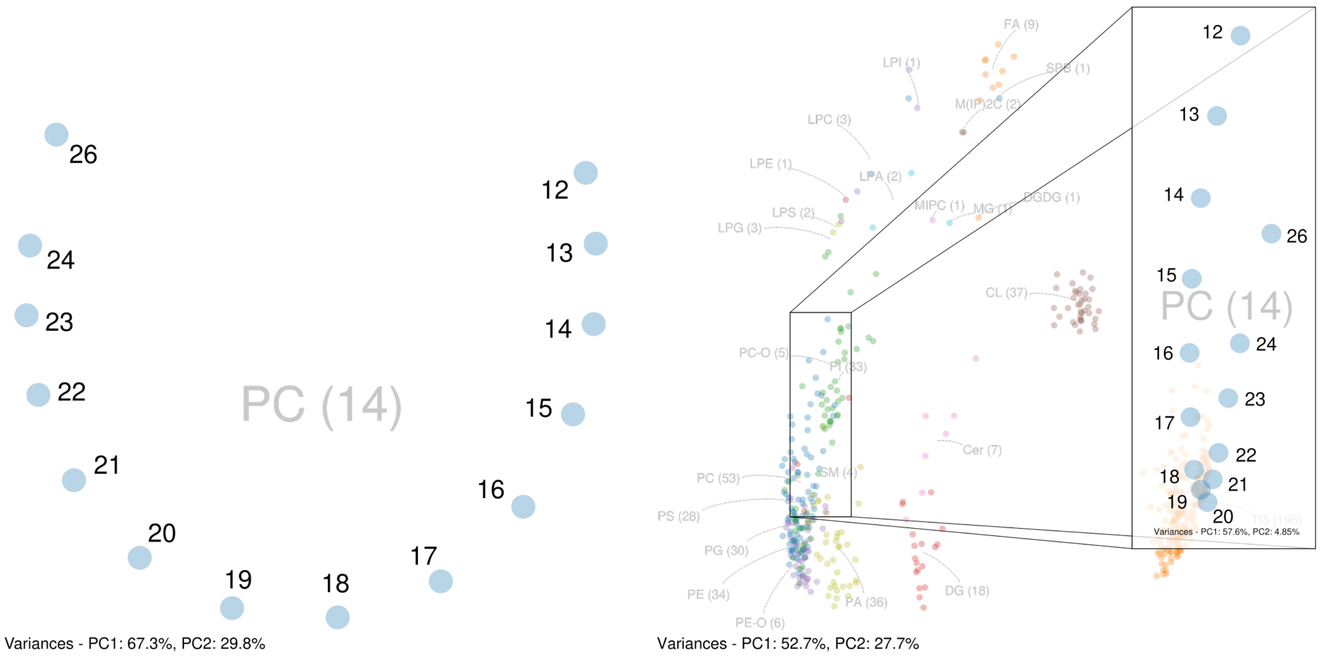

Figure S7: Preservation of the spatial organization of lipids in structural space model. Here, a list of 14 diacylglycerophosphocholine lipids PC 12:0/[12-24,26]:0 were analyzed individually (left) forming a sequential arc with increasing fatty acyl chains. On the right-hand side, the 14 lipids were analyzed along with a set of 500 lipids from different lipid categories. The 14 PC lipids preserve the sequential arc (magnified field) although slightly deformed. Please note that both the lipid diacylglycerophosphocholine and the principal components have the same abbreviation PC.

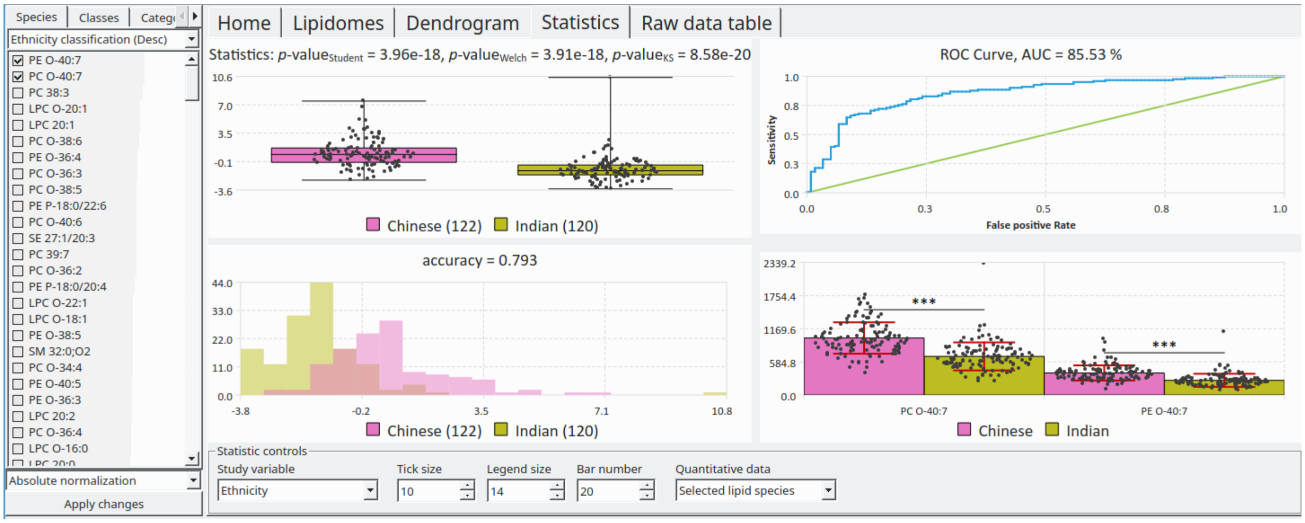

Figure S8: Reanalysis of a lipidomics study on human plasma. The results computed by LipidSpace are in agreement with the results reported by Saw et al. [2]. The lipid species PC O-40:7 and PE O-40:7 have the highest group separation potential.

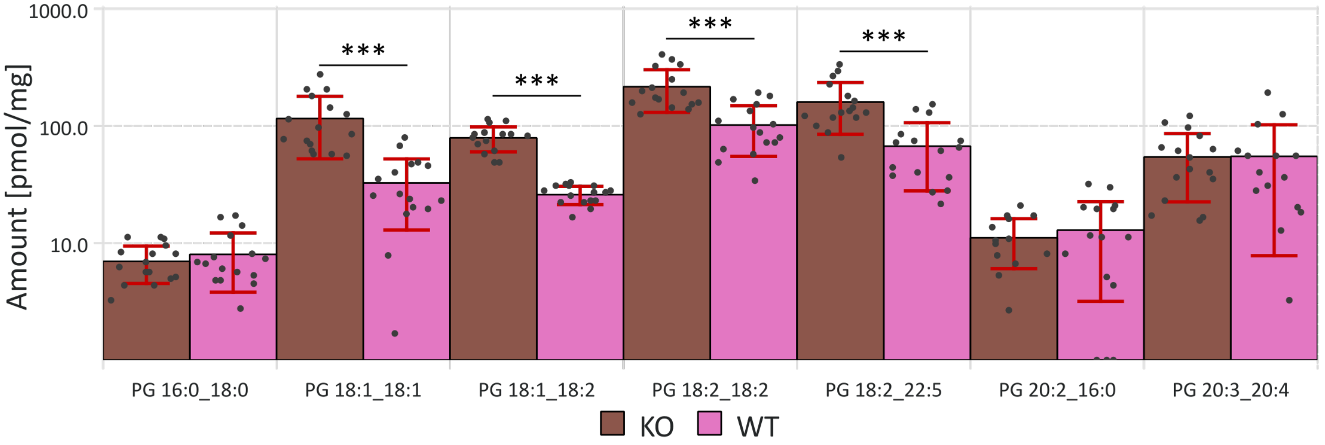

Figure S9: Reanalysis of a lipidomics study on mouse platelets. In the study of Peng et al. [3], the main abundant glycerophosphoglycerols (PG) species are significantly regulated between the conditions wild type (WT) and knockout (KO) with a p-value < 0.001.

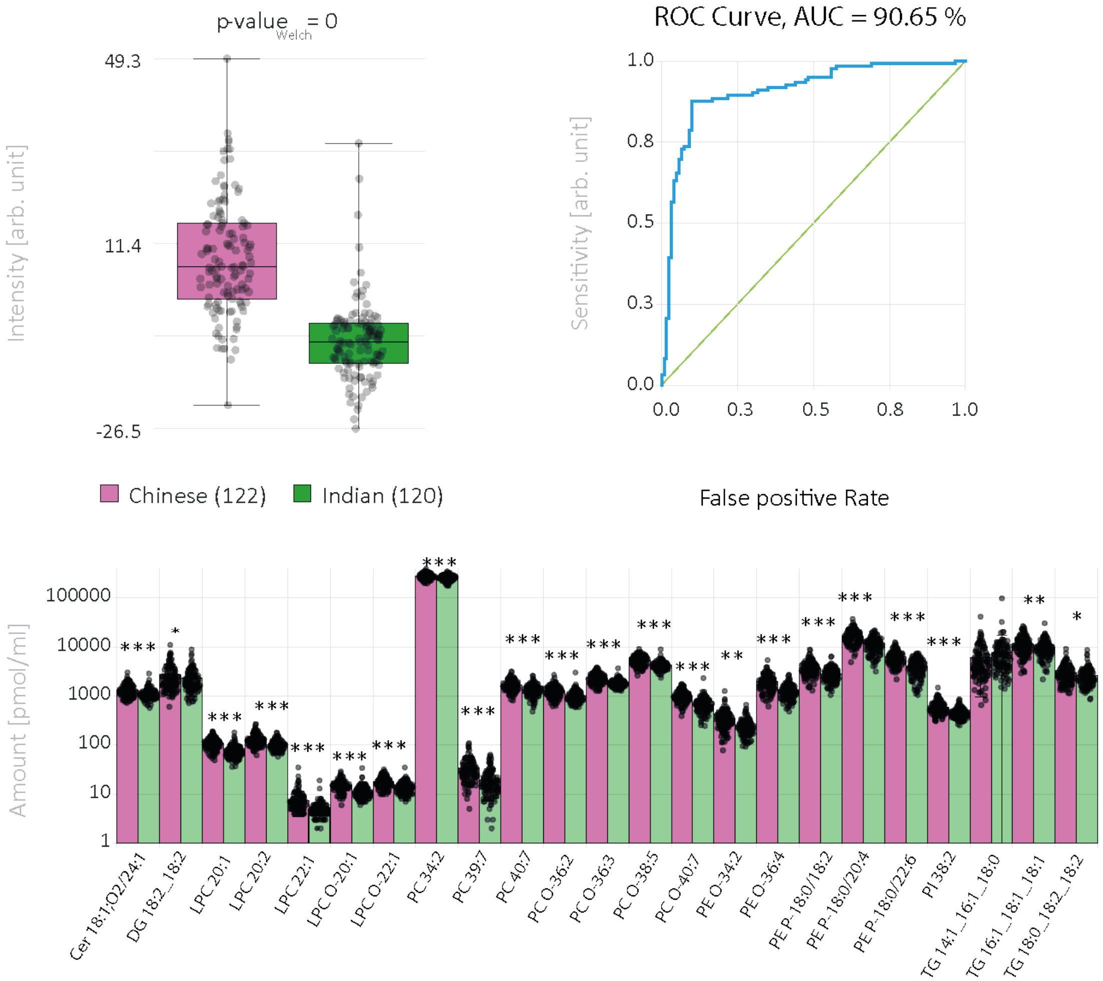

Figure S10: Study on different ethnicities. A separation model for the Chinese and Indian populations based on data derived from Saw et al. [2] was computed. Both populations were separated with an accuracy of 90.65 % under the consideration of a 23 lipids-comprising panel.

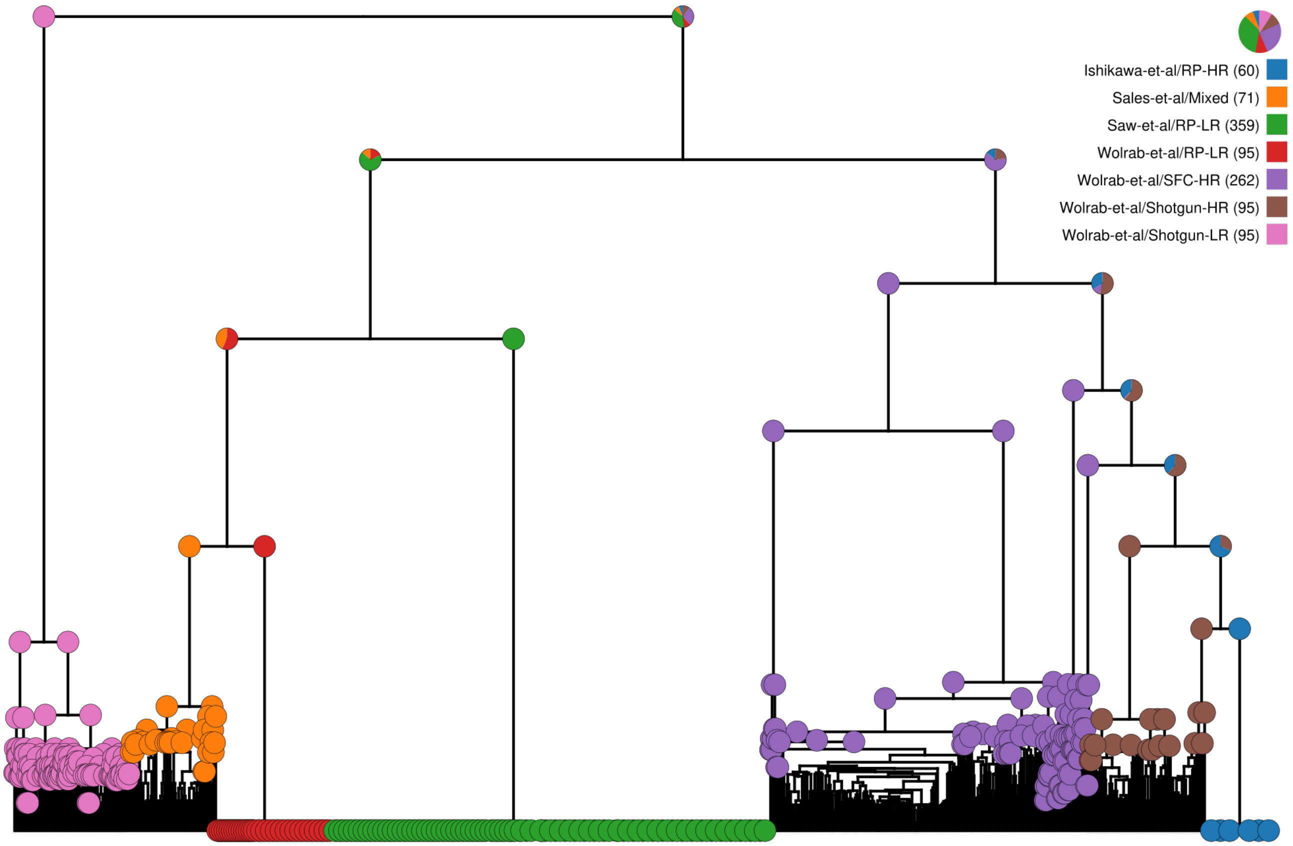

Figure S11: Comparison of seven different lipidomics experiments. A hierarchical clustering was performed by calculating the structural space between all 702 lipid species over all 1037 studies within seven datasets from four different lipidomics studies (Ishikawa et al. [4], Sales et al. [5], Saw et al. [2], Wolrab et al. [6]), and computing the pairwise distances between all lipidomes based on their corresponding lipid spaces. This clustering does not take the lipid abundances into consideration. Three studies (red, green, blue) have no further hierarchical structure within their branches because no differences in lipid composition were reported.

References

1 Benford, F. (1938) The law of anomalous numbers. Proc. Am. Philos. Soc. 78, 551-572.

2 Saw, W. Y. et al. (2017) Establishing multiple omics baselines for three Southeast Asian populations in the Singapore Integrative Omics Study. *Nat Commun*. **8**, 653.

3 Peng, B. et al. (2018) Identification of key lipids critical for platelet activation by comprehensive analysis of the platelet lipidome. *Blood*. **132**, e1-e12.

4 Ishikawa, M. et al. (2014) Plasma and serum lipidomics of healthy white adults shows characteristic profiles by subjects' gender and age. *PloS One*. **9**, e91806.

5 Sales, S. et al. (2016) Gender, Contraceptives and Individual Metabolic Predisposition Shape a Healthy Plasma Lipidome. *Sci Rep*. **14**, 27710.

6 Wolrab, D. et al. (2022) Lipidomic profiling of human serum enables detection of pancreatic cancer. *Nat Commun*. **13**, 124.
